# The Folded Radial Forearm Flap in Lip and Nose Reconstruction—Still a Unique Choice

**DOI:** 10.3390/jcm12113636

**Published:** 2023-05-24

**Authors:** Tobias Ettl, Maximilian Gottsauner, Thomas Kühnel, Michael Maurer, Johannes G. Schuderer, Steffen Spörl, Jürgen Taxis, Torsten E. Reichert, Mathias Fiedler, Johannes K. Meier

**Affiliations:** 1 Department of Oral and Maxillofacial Surgery, University Hospital Regensburg, Franz-Josef-Strauß-Allee 11, 93053 Regensburg, Germany; 2 Department of Otorhinolaryngology, University Hospital Regensburg, Franz-Josef-Strauß-Allee 11, 93053 Regensburg, Germany

**Keywords:** radial forearm flap, folded, lip, nose, flexible

## Abstract

(1) Background: The radial forearm flap (RFF) has evolved as the flap of choice for intraoral mucosal reconstructions, providing thin and pliable skin with a safe blood supply. Perforator flaps such as the anterolateral thigh (ALT) flap are increasingly being discussed for the same applications. (2) Methods: Patient history, treatment details, and outcome of 12 patents with moderate to extended defects of the lip and/or nose area that were reconstructed by a folded radial forearm flap were retrospectively evaluated for oncologic and functional outcomes. (3) Results: The mean oncologic and functional follow-up were 21.1 (min. 3.8; max. 83.3) and 31.2 (min. 6; max. 96) months, respectively. All flaps survived without revision. In eight cases, major lip defects were reconstructed by an RFF; in six patients, the palmaris longus tendon was included for lip suspension. The functional results in terms of eating, drinking, and mouth opening were good in five cases, while three patients were graded as fair due to moderate drooling. In seven cases, the major parts of the nose were reconstructed with two good and five fair (nostril constriction in three cases) functional results. (4) Conclusions: The folded RFF remains a unique free flap option for complex three-dimensional lip and nose reconstructions in terms of flexibility, versatility, and robustness.

## 1. Introduction

The reconstruction of maxillofacial defects by free flaps has evolved as the standard treatment during the last two decades. For many years, the radial forearm flap (RFF) has represented a major workhorse, due to its pliability enabling the restoration of thin, e.g., intraoral mucosal, defects [1,2]. The RFF was also described as a folded flap, in order to simultaneously replace both the skin and inner lining, e.g., in full-thickness cheek and lip defects [3,4].

The reconstruction of total or near-total lip defects is very demanding, as functional lips are a prerequisite for proper eating, drinking, and speaking. Moreover, lips represent an esthetic key structure in the face. Partial lip defects are, depending on size, mainly reconstructed using local flaps such as Abbé and Karapandzic or Johnson’s stair step technique, which at least partly restore orbicularis oris muscle function, thereby maintaining functional lip movement [5,6]. Subtotal or total defects, however, may not be restorable by local techniques, as mouth opening becomes too restricted, requiring free flap reconstruction [7].

The reconstruction of complex nasal defects means the reconstruction of skin, cartilage, and mucosa and poses a big challenge for the surgeon. Particularly, the restoration of the inner lining of large (subtotal) nose defects is one of the most demanding issues. While up to heminasal full-thickness defects may be lined by ipsilateral mucoperichondrial flaps based on the labial artery or by a three-staged folded forehead flap, a microvascular tissue transfer may become necessary for the lining of subtotal or total nasal defects [8,9,10]. The radial forearm flap has become the first option for this type of surgery [11].

In this study, we present a case series emphasizing the extraordinary versatility of the RFF in three-dimensional lip, nose, and mid face reconstruction and discuss the key surgical steps and drawbacks.

## 2. Materials and Methods

Ethical vote (Approval No. 18-1131-104) was obtained for this retrospective case review by the Institutional Ethical Committee. The reported patients were treated at the Department of Oral and Maxillofacial Surgery, Regensburg University Hospital, between the years 2015 and 2022. All data were retrieved from the charts. The inclusion criteria were patients treated for reconstruction of complex traumatic or oncologic defects involving the lip and/or nose with an RFF. Only RFFs that were folded were included. Only patients with a mean follow-up of at least 3 months were evaluated. Functional outcome for lip reconstruction was classified as follows: good eating and drinking is uneventfully possible with mouth opening of 3 cm; fair eating and drinking is possible with slight drooling or reduced mouth opening; poor eating or drinking is hardly possible because of massive drooling, missing lip control, or reduced mouth opening.

### Operative Technique

**Figure 1:**
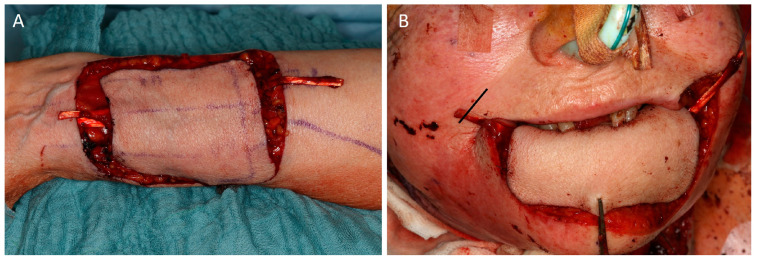
(Case 3) (**A**) Harvest of a radial forearm flap (RFF) with encased palmaris longus tendon (PLT). Distal flap harvest should leave enough space for adequate length of the PLT. (**B**) Reconstruction of the total lower lip. The PLT is tunneled below the skin and fixed to the zygomatic major muscle via a separate incision in the nasolabial fold (marking right side).

In all patients, the non-dominant arm was used for flap harvesting. In all patients, an Allen test was conducted before flap harvest. All RFFs were raised in a similar manner as previously described [1]. The skin island was distally placed on the forearm 2 cm away from the wrist flexor crease to lengthen the pedicle and was centered above the radial artery. Dissection started on the ulnar side and was performed in a subfascial plane toward the radial artery. In some cases, e.g., for nasal inner lining, dissection was started in a subcutaneous plane towards the flexor carpi radialis tendon. Care was taken to preserve the paratenon for uneventful donor wound healing. In six patients, a composite flap with encased palmaris longus tendon was harvested (Figure 1).

The distal cephalic vein was not continuously included in flap raising. Instead, pedicle dissection was carried high into the antecubital fossa and the coalition of the venae comitantes prior to the cephalic vein that was used for anastomosis. The radial artery was dissected up until it distally branches off from the brachial artery to the ulnar artery. During the whole dissection, the perfusion of the thumb was monitored by continuous pulse oximetry. After the flap was removed, the donor site was covered with a full-thickness skin graft from the inner upper-arm site. A bolster was tacked over the skin graft, and the arm was dressed and immobilized for 10 days. The flap was inset into the defect and fixed with some sutures. The pedicle was orientated through a subcutaneous tunnel into the neck for later anastomosis. In one case, the pedicle was tunneled to the pre-auricular area for anastomosis at the superficial temporal artery and vein. The most commonly used recipient vessels were the facial and superior thyroid artery and thyro-facial vein truncus. For anastomosis, all arteries were sewed with 8.0. For all venous anastomoses, a coupler was used. All anastomoses were performed in end-to-end fashion.

## 3. Results

Table 1 shows an overview of all the included patients.

**Figure 2:**
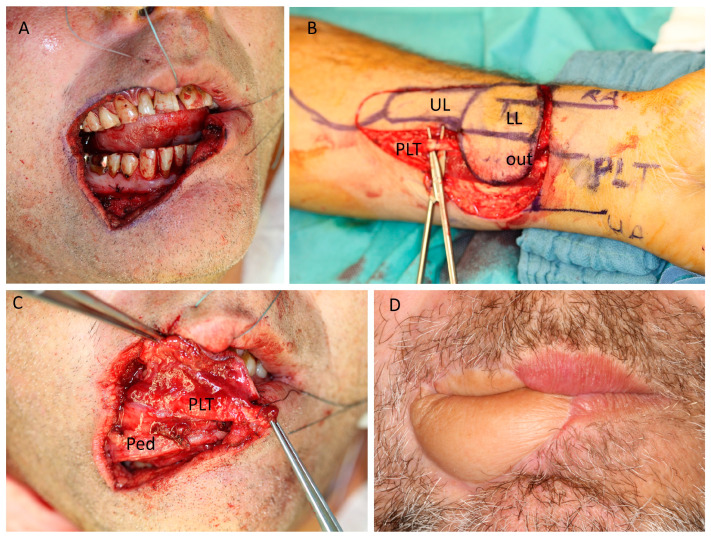
(Case 1) Lip reconstruction. Fifty-four-year-old male patient with melanoma of the lower lip and additional melanosis affecting 2/3 of the right lower lip and 1/3 of the right upper lip. (**A**) Intraoperative view after resection. (**B**) A 7 × 4 cm^2^ radial forearm flap (RFF), including the palmaris longus tendon (PLT) and a finger-shaped skin extension replacing the mucosa of the upper lip (UL), is raised. Lower lip (LL). Extraoral lip skin (Out). (**C**) Inset of the flap and reconstruction of the oral side. The vascular pedicle (Ped) is anastomosed to the facial artery and vein, and the palmaris longus tendon (PLT) is attached to the left remaining orbicularis oris muscle and the right zygomatic major muscle ensuring tension of the lower lip. (**D**) Extraoral and intraoral view after 9 months follow-up. Folding of the RFF enables the reconstruction of the internal and external parts of the lower lip.

**Figure 3:**
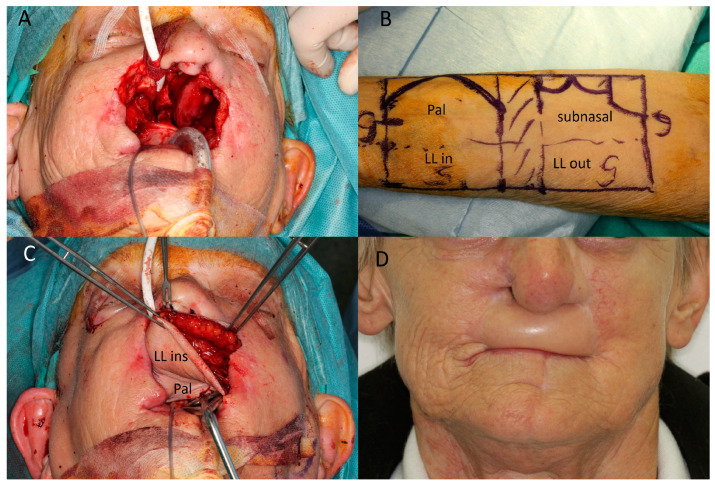
(Case 6) Lip and maxillary reconstruction. (**A**) Extensive resection of a basal cell carcinoma including anterior maxilla, upper lip, and nasal base. (**B**) Design of the radial forearm flap (RFF), overall 12 × 6 cm. Palatinal (pal). Lower lip (LL). Outside (out). Inside (in) (**C**) Inset of RFF with reconstruction of lip, anterior maxilla, and palate. (**D**) Post-op situation after 18 months. Recurrence at right ala was successfully treated with Vismodegib.

**Figure 4:**
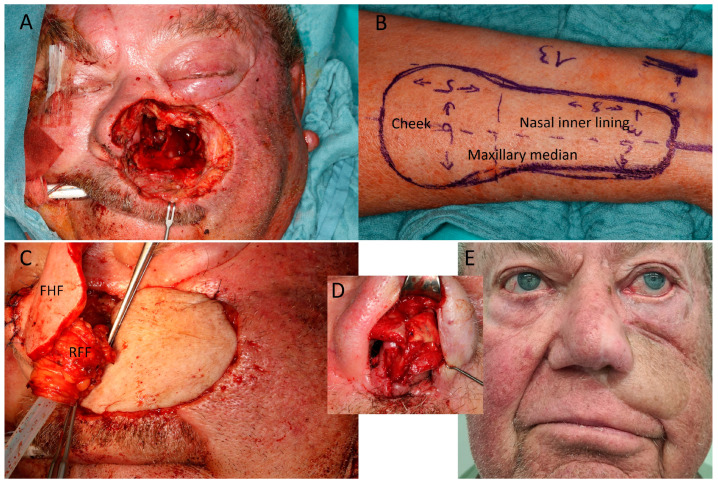
(Case 11) Mid face/nasal reconstruction after recurrent squamous cell carcinoma (SCC) of the left cheek and nose. (**A**) Defect including anterior septal base, left nasal ala, and anterior and median walls of maxillary sinus. (**B**) Design of the radial forearm flap (RFF), overall 13 × 6 cm. The width was 6 cm proximally and 3.5 cm distally (**C**) Inset of proximal RFF for cheek reconstruction; median RFF goes down to median wall of maxillary sinus. A titanium mesh restores the facial wall of the maxillary sinus. Turnover of distal RFF for nasal lining and suturing to split RFF edge as lateral nasal ala. Simultaneous forehead flap (FHF) due shortage of surgical capacities because of COVID-19 situation. (**D**) Reconstruction of left ala with ear cartilage 4 weeks later. (**E**) Appearance 15 months after alar cartilage framework and 12 months after radiotherapy.

**Figure 5:**
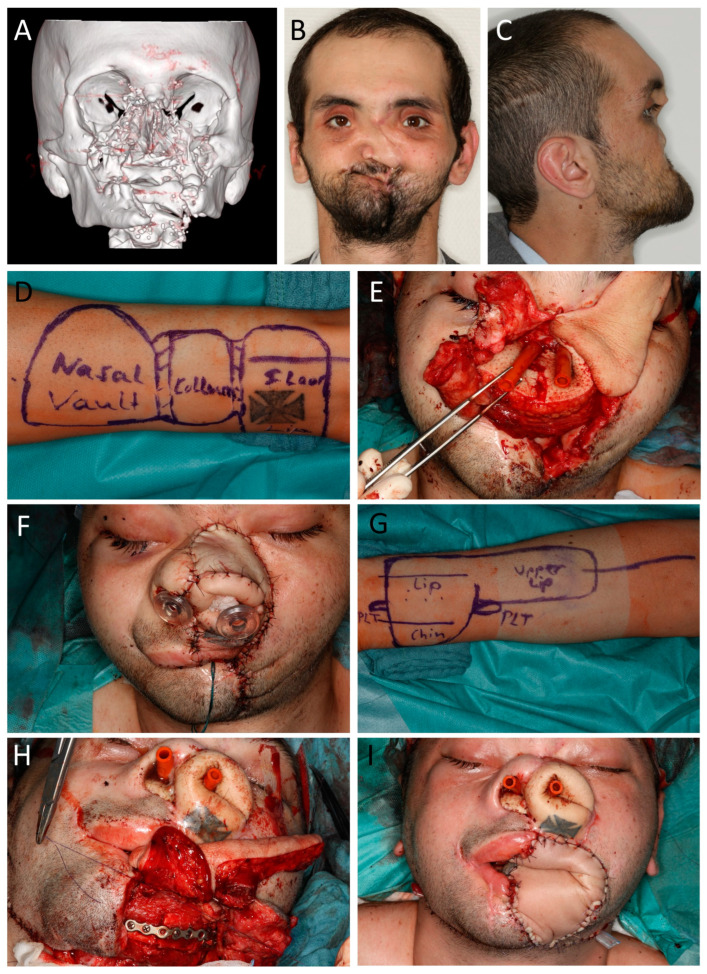
(Case 12) Mid and lower face reconstruction after avulsive gunshot injury. Patient initially showed up with tracheostomy and percutaneous gastral feeding tube. Additionally, he had a 4MRGN with pseudomonas aeruginosa, Citrobacter freundii, Klebsiella pneumoniae, and Staphylococcus aureus. Four surgeries were performed. Amoxicillin/Clavulanic acid was used for prophylaxis. (**A**) CT reconstruction shows the extensive destruction of the mid and lower face. (**B**,**C**) Pre-surgical view with missing mid face and no mouth opening. (**D**) Marking of the 14 × 6 cm^2^ radial forearm flap (RFF) from left arm for lining of nasal vault, columella, and upper lip (L out). (**E**) Inset of a free fibula flap (FFF) from the right leg with the skin island reconstructing the nasal floor and RFF starting laterally. The FFF skin paddle was split for columella lining and adaption with RFF. Both RFF and FFF were anastomosed to superficial temporal vessels (FFF right; RFF left). (**F**) End of first surgery with free skin graft on 360°-folded RFF for inner nasal lining and upper lip reconstruction. (**G**) Second surgery for reconstruction of the mandible and lip/commissure, restoring mouth opening. Marking of 7 × 14 cm^2^ RFF from right arm with palmaris longus tendon (PLT). (**H**) Inset of a FFF from the left leg. The skin paddle restores the anterior floor of mouth. Inset of the RFF with lining of the mucosal side first and suturing of the palmaris longus tendon to the remnant lower-right orbicularis oris and left zygomatic major muscle. Both free flaps were anastomized to the right and left facial arteries and veins. (**I**) End of surgery two.

**Figure 6:**
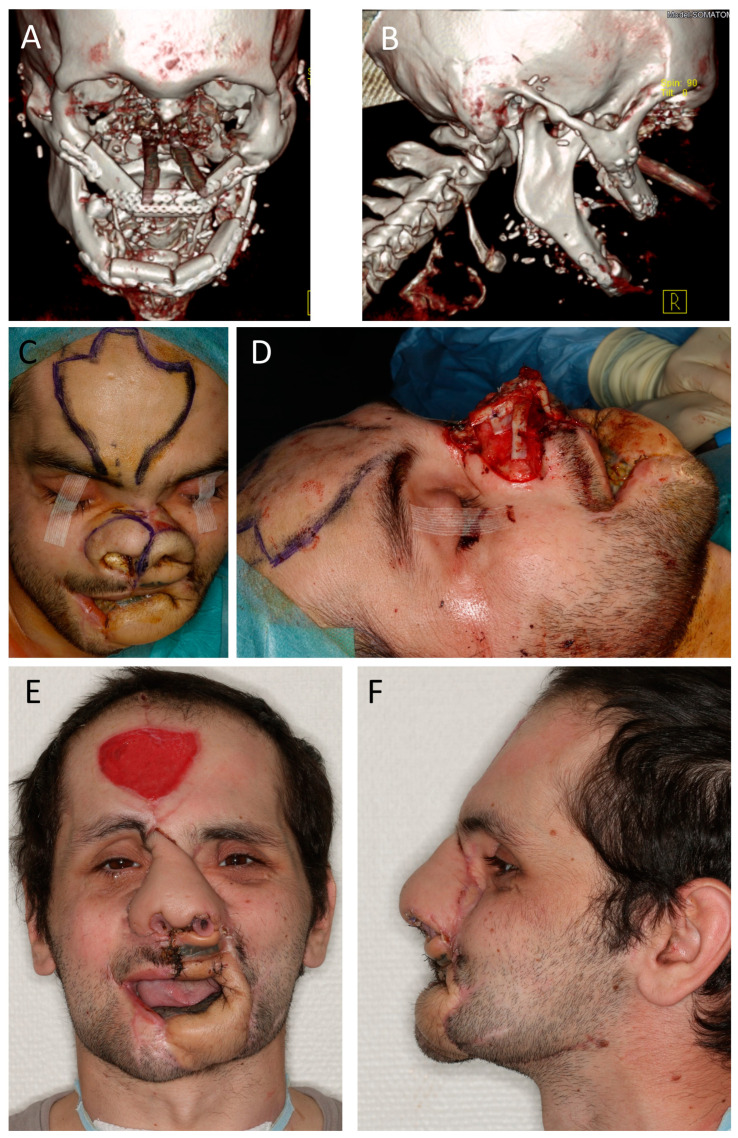
(Case 12, continued) Mid and lower face reconstruction. (**A**,**B**) CT scan showing bony fibula reconstruction of maxilla and mandible after steps 1 and 2. (**C**) Surgery three. Reconstruction of the skeletal base of the nose. Marking of the nasal hinge over flaps and the forehead flap. (**D**) Nasal framework reconstruction with iliac crest graft for osseous radix and rib graft for nose projection (columella, dorsum, and upper and lower lateral cartilage). (**E**,**F**) Situation 4 weeks after fourth surgery, showing projection of the mid face and good mouth opening. A wound-healing disturbance at the mid-upper lip was closed secondarily. The patient was able to eat, and the tracheostoma was removed on that day. After these steps, further refinements of the patient were denied by the government, and the patient was sent back to his home country.

Figure 2, Figure 3, Figure 4, Figure 5 and Figure 6 present four patients with five RFF reconstructions in more detail.

The final cohort consisted of 13 procedures in 12 patients (5 men; 7 women) where a folded or multi-island RFF was used (11 folded; 2 folded and multi-island) for lip and/or nose reconstruction (Table 1). One patient (case 12; Figure 5 and Figure 6) received two RFFs. The average patient age was 69 years (range: 27 years to 90 years). The main reason for surgery was a tumor condition. In detail, there were six squamous cell carcinomas (SCC), four basal cell carcinomas (BCC), and one melanoma. One patient (case 12; Figure 5 and Figure 6) underwent surgery as a result of a gunshot trauma. Five patients (cases 1, 3, 5, 8, and 11; mean dose 65.8 Gy) underwent postoperative radiotherapy of the head and neck area, and one patient (case 10) had preoperative radiotherapy. One patient (case 6) received vismodegib (Erivedge^®^, Roche, Basel, Switzerland) for post-surgical recurrence. The average flap size was 62.96 cm^2^ (range: 15 cm^2^ to 120 cm^2^). In six patients, the palmaris longus tendon was used for lip reconstruction (Figure 1 and Figure 2). The most commonly used recipient vessels were the facial (n = 10) and superior thyroid (n = 3) arteries and facial vein or thyro-facial vein truncus. In one patient (case 12), two additional fibula free flaps (FFF) were used for upper and lower jaw reconstruction (Figure 5 and Figure 6). There was 100% flap survival.

The oncologic and functional outcomes as well as the main issues during and after surgery are shown in Table 2.

The mean follow-up for all patients after RFF reconstruction was 21.1 (min. 3.8; max. 83.3) months. As some oncologic patients received RFF reconstruction secondarily after an ablative tumor resection, there was a different mean oncologic follow up of 31.2 (min. 6; max. 96) months. One flap (case 11; Figure 4C) showed intra-surgical venous congestion, after the initial flap inset when the upper end of the RFF was tacked down to restore the medial maxillary sinus wall. Congestion was immediately resolved after the release of the suture. All the patients with lip reconstructions were able to drink and eat via the oral route, although they were impaired by missing sensitivity and the different degrees of lip closure. With a more detailed view of the functional outcomes in this group, five patients were graded as good, with almost no impairment of oral intake, and three as fair, due to fluid drooling. One female patient (case 2), with a defect including the commissure, was suffering from drooling due to an undersized flap design. In one patient (case 3), the lower lip suspension weakened after radiotherapy, leading to slight drooling during drinking. One old patient (case 7; 90 years), with a large RFF restoring the main parts of the upper and lower lip, had difficulties in controlling lip competence while drinking. Lip esthetics were an issue in all lip reconstructions, as the vermillion color could never be restored.

The functional results after nasal reconstruction revealed a good result in two patients and a fair result in five cases (Table 2). In one elderly woman (case 8; 83 years), major parts of the anterior maxilla were missing, which led to the retraction of the upper lip with a loss of nasal projection. An additional fibula flap was refused by her relatives, and the maxillary denture could not be fixed properly on the remaining two teeth. In three cases (9,10, and 11), the reconstructed nostrils showed more or less constriction in the long term, impairing the nasal airway.

Five patients (cases 2, 4, 10, 11, and 12) had secondary surgeries for further refinements (Table 2). These were particularly necessary for complex nasal reconstructions concerning cartilage framework and tissue remodeling (Figure 4, Figure 5 and Figure 6). Two elderly patients (cases 7 and 9; 85 and 90 years) denied further refinements.

In regard to the donor site, there were no major complications. All the skin grafts healed regularly. No patients reported any mechanical impairments of their hand. Two patients claimed some hyposensitivity in the radial palm area.

## 4. Discussion

The radial forearm flap offers several advantages in maxillofacial, head, and neck reconstruction. It is easy to harvest and provides a constant anatomy with a long pedicle and large vessel diameters. The flap can be harvested as a composite flap that contains bone, brachioradialis muscle, sensory nerves, and tendons [1,12,13]. Its pliable, largely hairless skin with a rich and reliable vascularity allows for various three-dimensional designs for the reconstruction of the soft palate [14,15,16,17], providing the inner lining of the nose [10,11,18], the reconstruction of major lip defects [4,7], or bi-paddled flaps [3,19].

For flap folding, it is important that blood flow within the flap is not compromised. The authors observed that the RFF can uneventfully be folded along the vessel axis, as long as there is no direct kinking. Moreover, additional twisting in the perpendicular direction is possible with moderate tension (Figure 3).

During the last few years, several perforator flaps, particularly the anterolateral thigh flap (ALT), have extended the surgeons’ portfolio for soft tissue reconstruction and have also been reported to be used as folded flaps, e.g., for lip reconstructions [20,21]. The ALT flap has become very popular due to its low donor site morbidity and high versatility regarding the volume, size, and tissue compartments [22,23]. In addition, in our own institution, the ALT flap has replaced the RFF for many indications, e.g., for tongue or oropharyngeal reconstructions where more volume is necessary. However, from our point of view, the RFF seems more flexible and resistant compared to the anterolateral thigh flap. The fasciocutaneous perforator-based ALT flap does not tolerate folding as well as the RFF and can react with venous congestion. The problem of folding the ALT flap may be solved if bi-paddled flaps, based on separate perforators or even pedicles, are available [24]. Bi-paddled flaps were also described for the RFF, based on radial artery perforators [19,25]. The authors observed that serial skin paddles can be safely harvested without meticulous exposure of the perforators, as long as the skin paddle lies above the radial artery, due to its dense network of perforators [25]. For (sub)total lip reconstructions, particularly lower lip reconstructions, we would strongly recommend to harvest the palmaris longus tendon and anchor it to the remaining orbicularis oris muscle, modiolus, or zygomatic major muscle. This provides the necessary dynamic tension for drinking and eating, with just a little drooling or no drooling at all, enables oral opening, and preserves esthetics. A major issue, particularly with drooling, may rise when the RFF is bent over the commissure for upper and lower lip reconstruction. Although unfavorable in esthetics, care should be taken to supply enough flap bulk. The ALT flap seems to be an alternative for extensive lip reconstructions in non-obese patients [26]. For sufficient tension, a tensor fasciae latae sling graft should be inserted below the folded ALT flap [21]. Particularly in patients before or after radiotherapy, lip reconstruction with an RFF seems to be the most reliable compared to further alternatives such as the free gracilis flap, where the muscle structures, at least in our experience, bear the risk of detaching from the wound edges. Moreover, the gracilis flap is limited by the lack of skin and needs skin grafting.

The current case series contains seven cases where an RFF was performed for the restoration of different parts of the nasal inner lining. This technique is masterfully described in the works of Burget, Salibian, Menick, and colleagues [10,11]. Particularly for subtotal and total nasal defects, where major parts of the septum are missing, alternative techniques such as the three-staged folded or prelaminated forehead flap or the septal mucoperichondrial flap may no longer be applicable for the inner lining. In the authors’ opinion, the RFF remains the only free flap that reliably enables the thin rolling and folding needed to restore the lining of the septum, the nostrils, the dorsum, and the nasal floor. Apart from the favorable tissue composition and pliability, the RFF provides a long vascular pedicle to span the significant distance to the recipient vessels in the lateral neck, which is mandatory for nasal and mid face reconstructions with free flaps. Nevertheless, although a thin flap, the nose is far too bulky after RFF inner lining and requires secondary thinning, which is normally performed during surgical step 2 after 1–2 months and then repeated later. The pedicle must not be violated during this step. Another long-term concern is the neo-nostrils, which bear the risk of early constriction and stenosis and benefit, apart from a solid cartilage construction, from prolonged (6 weeks) nasal plugs (see Figure 6).

Of course, one should keep in mind that this treatment may regularly require four or more surgeries, while the patients involved are often of a very advanced age, as is apparent from this study. So the treatment must be carefully discussed with the patient and their relatives in advance, and, especially for elderly patients, a nose prosthesis may be an alternative.

## 5. Conclusions

In conclusion, the authors emphasize the still outstanding advantages of the radial forearm flap in terms of pliability, pedicle length, and safe vascular supply, which enable complex three-dimensional foldings for the restoration of complex facial structures such as the nose and lips. Designing different skin paddles enables the reconstruction of multi-located defects.

## Figures and Tables

**Table 1:** Patient and treatment overview.

Pat	Sex	Age (Years)	Defect	Reason	Size	PLT Used	Recepient Vessels	Further Flaps	Radiotherapy
1	M	54	Partial lower, upper lip, commissure, oral cavity	Melanoma	7 × 4	Yes	FA and vein	No	Post RT
2	W	72	Partial lower, upper lip, commissure, cheek	SCC	7 × 4	Yes	FA and vein	No	No
3	W	79	Total lower lip, chin	SCC	8 × 6	Yes	FA and vein	No	Post RT
4	M	75	Partial lower, upper lip, commissure, cheek	BCC	6 × 4	Yes	FA and vein	No	No
5	W	73	Subtotal upper lip, commissure	SCC	6 × 4	No	FA and vein	No	Post RT
6	W	61	Maxilla, total upper lip, nasal base	BCC	12 × 6	No	STA and vein	No	No
7	M	90	Maxilla, nose, total upper and partial lower lip	SCC	15 × 8	Yes	FA and vein	FHF	No
8	W	83	Anterior maxilla, nasal base	SCC	9 × 8	No	FA and vein	No	Post RT
9	W	85	Cheek, nose	BCC	10 × 8	No	FA and vein	No	No
10	W	74	Maxilla, nose	BCC	11 × 7	No	STA and facial vein	FHF	Pre RT
11	M	81	Cheek, nose	SCC	13 × 6	No	FA and thyreofacial trunk	FHF	Post RT
12	M	27	Mid and lower face, nose, lips	Trauma	14 × 6 and 14 × 7	Yes	STA and vein; FA and vein	2× FFF, FHF, rib, iliac crest	n/a

Abbreviations: BCC, basal cell carcinoma; SCC, squamous cell carcinoma; FA, facial artery; STA, superior thyroid artery; FFF, fibula free flap; PLT, palmaris longus tendon; RFFF, radial forearm free flap; FHF, forehead flap; n/a, not applicable.

**Table 2:** Patient outcomes.

Pat	Oncologic Outcome	Functional Outcome/Issues	Secondary Procedures
1	Death after 29 months due to distant metastasis	Good	None
2	FOR after 8 months	Fair, drooling right commissure, flap too small	Vestibuloplasty, cheek advancement after 4 months
3	FOR after 12 months	Good, slim lip, little loss of tension	None
4	FOR after 16 months	Good	Cheek thinning after 5 months
5	Death due to secondary tumor after 6 years	Good	Maxillary prosthesis after 3 months
6	Recurrence after 8 months; lost to follow-up after 4 years	Good, dehiscence of right ala due to tumor recurrence	None, persistent small defect of right nasal ala
7	Non-tumor-related death after 14 months	Fair, insufficient lip competence control	None, further surgery refused
8	Lost to follow-up after 6 months	Fair, flap retraction due to maxillary deficiency	Partial denture for lip advancement
9	FOR after 20 months	Fair, nostril constriction	None, further surgery refused
10	FOR after 8 years	Fair, flap shrinkage, nostrils’ constriction	FHF, rib graft after 4 months, flap thinning after 7 months
11	FOR after 22 months	Good, slight ectropium, left nostril smaller	Thinning FHF, ear cartilage grafting after 4 weeks, lateral canthoplasty
12	n/a	Fair, infection, color esthetics	Rib graft, iliac bone graft, FHF, thinning FHF (captions of Figure 5 and Figure 6 for details)

Abbreviations: FOR, free of recurrence; n/a, not applicable; FHF, forehead flap.

## Data Availability

Data can be obtained from the authors that conducted the work, independently from the industry, on request. Data are not stored on publicly available servers.
